# Prospective trial to evaluate the prognostic value of different nutritional assessment scores for survival in pancreatic ductal adenocarcinoma (NURIMAS Pancreas SURVIVAL)

**DOI:** 10.1002/jcsm.12796

**Published:** 2021-09-20

**Authors:** Max Heckler, Ulla Klaiber, Felix J. Hüttner, Sebastian Haller, Thomas Hank, Henrik Nienhüser, Philip Knebel, Markus K. Diener, Thilo Hackert, Markus W. Büchler, Pascal Probst

**Affiliations:** ^1^ Department of General, Visceral and Transplantation Surgery University of Heidelberg Heidelberg Germany

**Keywords:** Malnutrition, Oncology, Pancreatic cancer, Surgery, Clinical trial

## Abstract

**Background:**

Malnutrition is associated with poor survival in pancreatic cancer patients. Nutritional scores show great heterogeneity diagnosing malnutrition. The aim of this study was to find the score best suitable to identify patients with malnutrition related to worse survival after surgery for pancreatic ductal adenocarcinoma (PDAC). This study represents a follow‐up study to the prospective NURIMAS Pancreas trial that evaluated short term impact of nutritional score results after surgery.

**Methods:**

Risk of malnutrition was evaluated preoperatively using 12 nutritional assessment scores. Patients were followed‐up prospectively for at least 3 years. Patients at risk for malnutrition were compared with those not at risk according to each score using Kaplan–Meier survival statistics.

**Results:**

A total of 116 patients receiving a PDAC resection in curative intent were included. Malnutrition according to the Subjective Global Assessment score (SGA), the Short Nutritional Assessment Questionnaire (SNAQ), and the INSYST2 score was associated with worse overall survival (SGA: at‐risk: 392 days; not at‐risk: 942 days; *P* = 0.001; SNAQ: at‐risk: 508 days; not at‐risk: 971 days; *P* = 0.027; INSYST2: at‐risk: 538 days; not at risk: 1068; *P* = 0.049). In the multivariate analysis, SGA (hazard ratio of death 2.16, 95% confidence interval 1.34–3.47, *P* = 0.002) was associated with worse overall survival.

**Conclusions:**

Malnutrition as defined by the Subjective Global Assessment is independently associated with worse survival in resected PDAC patients. The SGA should be used to stratify PDAC patients in clinical studies. Severely malnourished patients according to the SGA profit from intensified nutritional therapy should be evaluated in a randomized controlled trial.

## Background

Pancreatic ductal adenocarcinoma (PDAC) is one of the deadliest malignancies, and surgical resection—usually within multimodal treatment strategies—still represents the only potential cure. Malnutrition and cachexia are characteristic for PDAC and affect the course of the disease and its prognosis, particularly after curative resection.^1^, ^2^, ^3^ A variety of different nutritional assessment scores are available to clinicians. However, comprehensive validation of these tools is still lacking for many diseases, including PDAC. The prospective *Nutritional Risk in Major Abdominal Surgery (NURIMAS) Pancreas* study^4^ was conducted to identify the scores with the best predictive performance with regards to major surgical complications. Two major conclusions were drawn from the initial study: first, the 12 scores defined the malnourished population drastically different. For example, the Nutritional Risk Index identified about 1% of the population ‘at risk’, while the Nutritional Risk Classification identified about 80% of the population ‘at risk’. Second, none of the included scores defined malnourished patients that were prone to more major complications. However, the NURIMAS Pancreas trial did not assess long‐term oncological outcomes.

The aim of this study was to investigate 12 available nutritional assessment scores for potential associations with overall survival (OS) and recurrence‐free survival (RFS) after resection for PDAC.

## Methods

This study is a long‐term follow up of the prospective NURIMAS pancreas study (registration number in the German Clinical Trials Registry: DRKS00006340).^4^ The study and the follow‐up amendment were approved by the ethics committee of the medical faculty at Heidelberg University (S‐170/2014). The study protocol was published before publication of the initial manuscript.^5^

### Patient selection

Consecutive patients scheduled for pancreatic surgery at the Department of General, Visceral and Transplantation Surgery, University of Heidelberg, Germany, were screened for eligibility. Inclusion criteria were informed written consent, age between 18 and 90 years, and no history of previous pancreatic resection. In this long‐term analysis of the NURIMAS Pancreas study, only those patients with histologically confirmed PDAC were included.

### Nutritional assessment scores

Nutritional parameters were assessed within 36 h before surgery. The following nutritional scoring indices were included: Nutritional Risk Index (NRI)^6^; Nutritional Risk Screening Score (NRS)^7^ and Nutritional Risk Screening Score 2002 (NRS 2002)^8^; Subjective Global Assessment (SGA)^9^; Malnutrition Universal Screening Tool (MUST)^10^; Mini Nutritional Assessment (MNA) and Mini Nutritional Assessment Short Form,^11^ Short Nutritional Assessment Questionnaire (SNAQ)^12^; Imperial Nutritional Screening System 1 and Imperial Nutritional Screening System 2 (INSYST)^13^; European Society for Clinical Nutrition and Metabolism.^14^ Information on the items included in each nutritional assessment score is given in Table S1. For scores with more than two modes, only those assigned to the highest‐risk class were defined as being ‘at risk for malnutrition’ while all other categories were classified as ‘not at risk for malnutrition’.

### Outcome assessment

For the assessment of long‐term outcomes, an additional study visit was performed 3 years after index operation. Patients and/or their general practitioners were contacted for survival status. OS and RFS were assessed. For OS, death due to any cause after the index operation was considered. For RFS, recurrence was defined as any radiological recurrence, local, or distant.

### Statistical analysis

Overall survival and RFS were calculated from the time of surgery. Kaplan–Meier survival analysis was performed to compare OS and RFS. Hazard ratio (HR) of death was computed for each score (3 year survival). Cox proportional hazard ratio was used for multivariate assessment. Risk scores that were significantly associated with OS in univariate analysis were separately assessed in a cox model. Other preoperative risk factors were included in the model such as age, neoadjuvant chemotherapy and American Society of Anesthesiologists performance status. No postoperative risk factors, for example, adjuvant chemotherapy, were included in the model. For multivariate analysis missing information was imputed by the Fisher's optimum scoring method.^15^ Continuous variables were dichotomized for the regression model. Statistical analysis was performed using the open source software R (V3.3) and the hmisc and survminer packages.

## Results

### Patients characteristics

**Figure 1:**
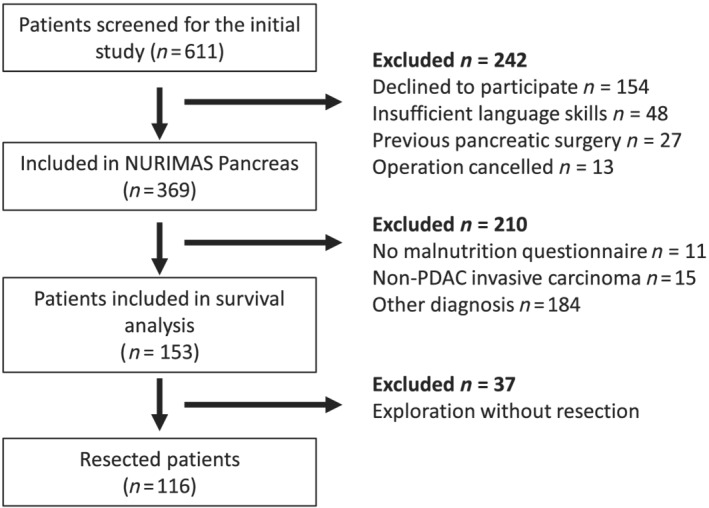
Patient flow.

From August 2014 to July 2015, 369 patients were recruited in the NURIMAS Pancreas study. All patients were screened for their final histopathologic diagnosis. Those patients with PDAC (*n* = 153) were further screened. In 116 patients, a resection was completed, whereas in 37 patients, surgery was aborted due to advanced disease. All 116 patients with completed resection were followed up until at least 3 years after resection, and no censoring was required. Patient flow is depicted in *Figure* *1*. Median age was 67 years, 62 patients (53.4%) were female patients. Baseline characteristics of included patients are displayed in *Table* *1*.

**Table 1:** Patient baseline data

Patients characteristics	
*n*	116
Age (years)^a^	65.05 (11.01)
Gender (female)^b^	62 (53.4)
Weight loss (kg)^a^	5.49 (5.26)
ASA status^b^	
1	3 (2.6)
2	66 (56.9)
3	47 (40.5)
AJCC^2^	
IA	3 (2.7)
IB	2 (1.8)
IIA	16 (14.2)
IIB	71 (62.8)
III	3 (2.7)
IV	18 (15.9)
Diabetes mellitus^b^	
None	87 (75.0)
NIDDM	13 (11.2)
IDDM	16 (13.8)
Duration of surgery (min)^a^	301.01 (104.42)
Blood loss (mL)^a^	687.47 (764.93)

AJCC, American Joint Committee of Cancer 7th edition; ASA, American Society of Anesthesiologists; IDDM, insulin‐dependent diabetes mellitus; NIDDM, non‐insulin‐dependent diabetes mellitus.

^a^ Presented as mean (standard deviation in brackets).

^b^ Presented as total number (% in brackets).

### Nutritional risk scores and overall survival after resection for pancreatic ductal adenocarcinoma

Kaplan–Meier survival analysis was performed for all available scores. Risk groups were defined according to score results. Nutritional Risk Classification^16^ was excluded from further analysis, because all patients were classified as ‘at risk’ per definition of the score.

**Figure 2:**
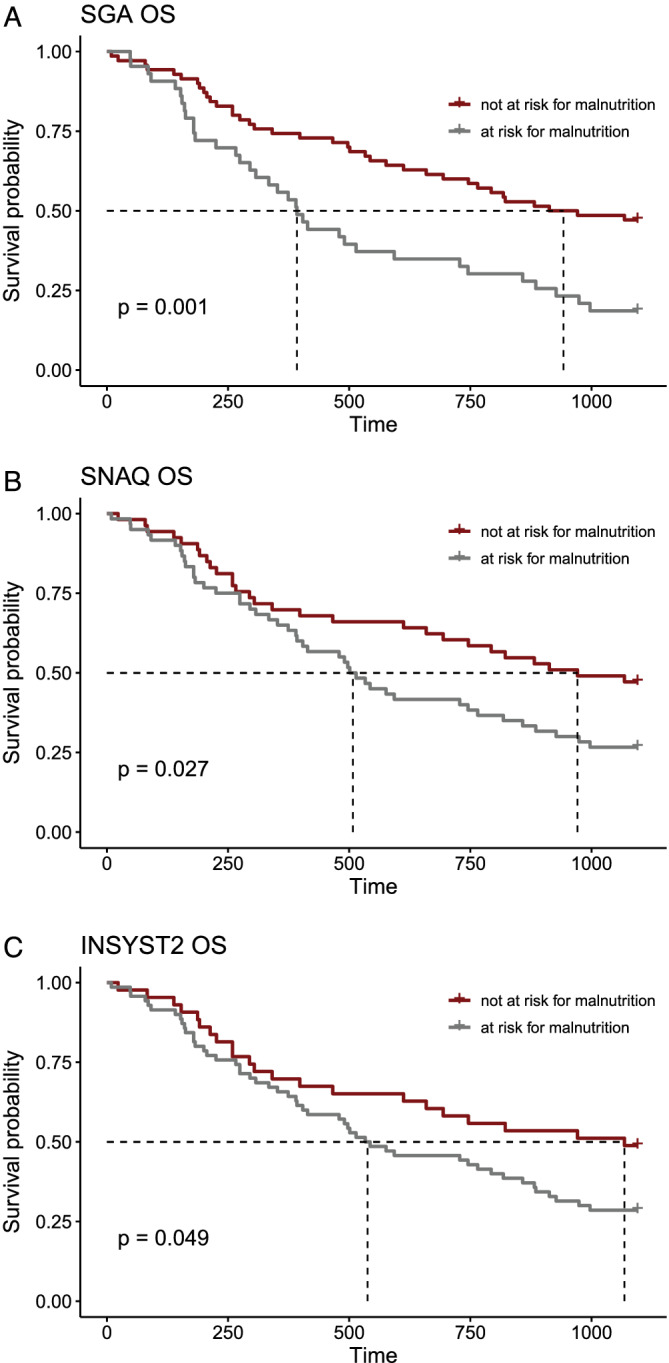
Kaplan–Meier overall survival curves for SGA (*A*), SNAQ (*B*), and INSYST2 (*C*). INSYST2, Imperial Nutritional Screening System 2; OS, overall survival; SGA, Subjective Global Assessment; SNAQ, Short Nutritional Assessment Questionnaire.

The following results focus on the 116 patients that were successfully resected. Malnutrition according to the SGA score, the Short Nutritional Assessment Questionnaire (SNAQ) and the INSYST2 was associated with shorter median OS compared with the group with a normal nutritional status as defined by the respective scores (SGA: at risk for malnutrition: 392 days [95% confidence interval (CI) 295–745 days]; not at risk for malnutrition: 942 days (95% CI 694–NA days); *P* = 0.001; *Figure* *2A*; SNAQ: at risk for malnutrition: 508 days (95% CI 392–818 days); not at risk for malnutrition: 971 days (95% CI 694–NA days); *P* = 0.027; *Figure* *2B*; INSYST2: at risk: 538 days (95% CI 404–882 days); not at risk: 1068 days (95% CI 612–NA days); (*P* = 0.049). Malnutrition as defined by SGA and SNAQ scores was additionally associated with an elevated HR of death within a 3 year time interval (SGA: HR 2.17, CI 1.37–3.47; *P* < 0.001; SNAQ: HR 1.68, CI 1.06–2.73; *P* = 0.03) in univariate analysis. The INSYST2 had no significantly elevated HR of death in this analysis (HR 1.65, CI 0.998–2.73; *P* = 0.05). None of the other included scores significantly correlated with OS (*Table* *2*).

**Table 2:** Detailed results of overall survival according for each nutritional assessment score

Score	At risk for malnutrition (out of 116)	OS not at risk for malnutrition (days)	OS at risk for malnutrition (days)	HR of death	95% CI	Logrank, *P* value
Nutritional Risk Index (NRI)	25 (22 %)	756	414	1.34	0.78–2.31	0.3
Nutritional Risk Screening Score (NRS)	102 (88%)	466	755	0.67	0.35–1.33	0.3
Nutritional Risk Screening Score 2002 (NRS2002)	90 (78%)	852	593	1.45	0.81–2.59	0.2
Subjective Global Assessment (SGA)	43 (37%)	**942**	**392**	**2.17**	**1.37–3.47**	**<0.001**
Malnutrition Universal Screening Tool (MUST)	63 (54%)	779	576	1.36	0.85–2.21	0.2
Mini Nutritional Assessment (MNA)	80 (69%)	769	694	1.03	0.62–1.72	0.9
Mini Nutritional Assessment Short Form (MNASF)	107 (92%)	398	756	0.53	0.25–1.11	0.09
Short Nutritional Assessment Questionnaire (SNAQ)	60 (52%)	**971**	**508**	**1.70**	**1.00–2.73**	**0.03**
Imperial Nutritional Screening System 1 (INSYST1)	100 (86%)	694	736	0.94	0.48–1.83	0.8
Imperial Nutritional Screening System 2 (INSYST2)	71 (61%)	1068	538	1.65	0.99–2.73	0.05
ESPEN malnutrition criteria (ESPEN)	37 (32%)	779	497	1.37	0.85–2.21	0.2

### Recurrence‐free survival in relation to nutritional status

**Figure 3:**
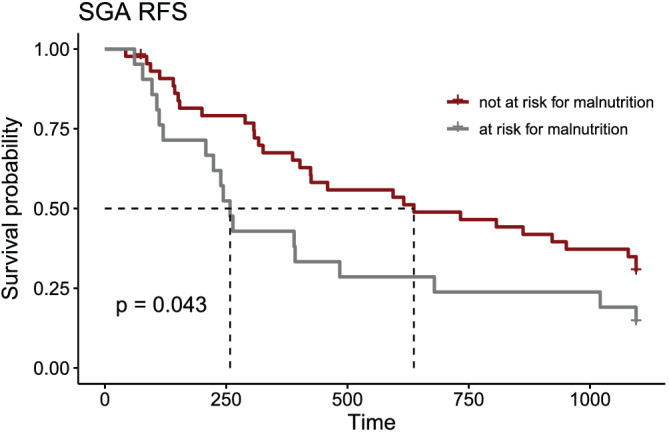
Kaplan–Meier recurrence‐free survival curve for SGA. RFS, recurrence‐free survival; SGA, Subjective Global Assessment.

Recurrence‐free survival was significantly shorter in patients at risk according to the SGA compared with normal nutritional status [at risk for malnutrition: 258 days (95% CI 208–1021 days); not at risk for malnutrition: 637 days (95% CI 402–1095 days); *P* = 0.043; *Figure* *3*]. All other scores showed no significant association with RFS (data not shown).

Hazard ratio of recurrence within a 3 year interval after surgery was elevated in patients with malnutrition according to SGA (HR 1.82, CI 1.01–3.28, *P* = 0.04). None of the other scores predicted RFS (data not shown).

### Multivariate analysis

**Figure 4:**
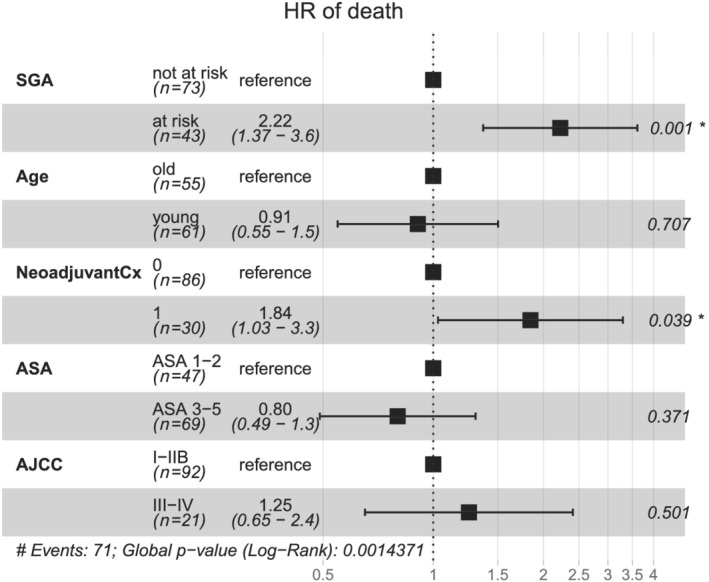
Multivariate analysis of overall survival. Hazard ratio of death. Age: 65 years age was used as a cut‐off for young/old. AJCC, American Joint Committee of Cancer 7th edition; ASA, American Society of Anesthesiologists; NeoadjuvantCx, neoadjuvant chemotherapy (1 = yes, 0 = no); SGA, Subjective Global Assessment.

In a multivariate cox model including age, American Society of Anesthesiologists performance status, neoadjuvant chemotherapy, and AJCC stage as factors, malnutrition according to the SGA score was associated with an elevated HR of death (2.22, 95% CI 1.37–3.6, *P* = 0.001; *Figure* *4*). Neoadjuvant chemotherapy was associated with an increased HR of death (HR 1.84; 95% CI 1.03–3.3; *P* = 0.039). SNAQ and INSYST2 scores were not significantly associated with an elevated HR in this model (data not shown).

## Discussion

Cachexia and malnutrition are characteristic for PDAC and are associated with a poor prognosis.^1^ In order to define and quantify malnutrition a plethora of scores has been developed over the past decades. However, prospective validation by means of relevant outcomes is still lacking for pancreatic surgery. Nutritional scores in the context of abdominal surgery have special requirements. Scores that automatically put patients at risk of malnutrition when they receive surgery and/or have cancer will not provide valuable information and overestimate the number at risk substantially. On the other hand, scores that underestimate malnutrition are of no value either. A meaningful score therefore needs to discriminate, ideally, while being derived out of easily accessible information.

The initial NURIMAS Pancreas study has established that a high variance among nutritional scores exists and that none of the tested scores reliably predicts major surgical complications.^4^ In the present follow‐up study, the SGA score predicts long‐term survival after surgery for PDAC. These results indicate that the SGA score might represent a valid tool to identify a population with elevated risk of relapse and death after PDAC resection. The definitive causal relationship between elevated SGA score and reduced survival can only be speculated on at this point: the NURIMAS Pancreas study showed no elevated complications (including in‐hospital death) in patients classified as malnourished by SGA score. This indicates that ‘at‐risk for malnutrition’ patients might be more susceptible for early metastasis or early local recurrence due to a malnourished and potentially immunocompromised state. Alternatively, the malnourished state assessed pre‐surgery might reflect a higher subclinical tumour burden at baseline. This is in line with the finding of the multivariate analysis, which showed an elevated HR of death in patients receiving neoadjuvant chemotherapy—probably due to a more advanced stage of the disease as assessed by pre‐operative cross‐sectional imaging and CA 19‐9 levels. Another intriguing option is that patients with an at‐risk status according to the SGA score are less likely to receive adjuvant chemotherapy and therefore have a limited survival: adjuvant chemotherapy is routinely recommended after resection of PDAC and is significantly associated with an improved survival.^17^, ^18^ Reduced clinical condition and nutritional state might prevent patients from receiving chemotherapy, hence worsening their prognosis.^19^ Only per‐operative items were included in our multivariate analysis to reduce the risk of bias; hence, the relationship between SGA result and completion of adjuvant chemotherapy needs to be addressed in additional studies. However, the SGA score might represent a tool to identify these malnourished patients in an early stage and offer an opportunity to intervene. Frequent nutritional monitoring and therapy with the goal of an adequate caloric intake could be an option to improve access to chemotherapy and ultimately prolong survival. Further prospective studies are urgently needed to elucidate the association between reduced SGA score and worse survival found here and to investigate the potential of SGA guided nutritional interventions.

Interestingly, the SGA score includes only items derived from patient history and clinical examination: weight loss, nutritional intake, gastrointestinal symptoms, fatigue, metabolic requirement and assessment of oedemas and muscle/fat loss. It does not require laboratory parameters or data from cross sectional imaging.

New tools for nutritional assessment are continuously developed and validated. It is especially important to acknowledge that the SGA score has a patient generated modification. This interesting tool might be even more convenient in the context of pre‐operative assessment; however, the score was not included in the original list of scoring systems evaluated in this study. Further prospective studies should be conducted to assess this important variation of the SGA score in the context of pancreatic cancer.

While being a prospective study, including a high number of patients recruited within a relatively short time period, it has some limitations: the study was conducted in a single high‐volume centre, resulting in potential selection bias. While a multivariate analysis was conducted, it is possible that unknown confounders were missed. As discussed in the limitations of the original NURIMAS Pancreas publication,^4^ sample‐size calculation was based on an assumed prevalence of malnutrition of 70%. Only some scores reached a prevalence of 70%, rendering the others susceptible to potential type B error, falsely classifying malnourished patients as not malnourished.

The present study focuses on pre‐operative nutritional assessment, which only represents one aspect of perioperative nutrition of cancer patients. Post‐operative assessment is another important pillar and should be assessed in further trials. We propose structured evaluation of total perioperative nutritional monitoring in cancer patients in large prospective trials.

Previous studies investigating the impact of nutritional status on the outcome of pancreatic surgery were either small‐sized, retrospective and/or selectively investigated one score only. NURIMAS Pancreas was designed and conducted to comprehensively assess eligible scores/indices in a sufficiently numbered, prospective study.^20^, ^21^, ^22^, ^23^ One key finding of the initial NURIMAS Pancreas study was that short‐term surgical complications are not more frequent in patients that were classified as malnourished by any of the included scores. Therefore, an at‐risk status for malnutrition should not result in a major delay of resection. The present study demonstrates that long‐term outcomes differ in at‐risk for malnutrition and not‐at‐risk for malnutrition populations according to the SGA score. This emphasizes the need for long‐term follow up of at‐risk patients, while the notion of the initial study still holds true.

In summary, nutrition status determined by SGA score is associated with survival after surgery for PDAC. It might aid in the stratification of PDAC patients receiving surgery. Further studies are needed to test potential therapeutic consequences.

## Conflict of interest

All authors declare that they have no conflict of interest.

## Funding

No extramural funding was used.

## Supporting information


**Table S1.** Items included in nutritional assessment scores.

## References

[1] Bachmann J , Heiligensetzer M , Krakowski‐Roosen H , Büchler MW , Friess H , Martignoni ME . Cachexia worsens prognosis in patients with resectable pancreatic cancer. J Gastrointest Surg 2008;12:1193–1201. 18347879 10.1007/s11605-008-0505-z

[2] Bachmann J , Ketterer K , Marsch C , Fechtner K , Krakowski‐Roosen H , Büchler MW , et al. Pancreatic cancer related cachexia: influence on metabolism and correlation to weight loss and pulmonary function. BMC Cancer 2009;9:255. 19635171 10.1186/1471-2407-9-255 PMC2741486

[3] Kays JK , Shahda S , Stanley M , Bell TM , O'Neill BH , Kohli MD , et al. Three cachexia phenotypes and the impact of fat‐only loss on survival in FOLFIRINOX therapy for pancreatic cancer. J Cachexia Sarcopenia Muscle 2018;9:673–684. 29978562 10.1002/jcsm.12307 PMC6104116

[4] Probst P , Haller S , Bruckner T , Ulrich A , Strobel O , Hackert T , et al. Prospective trial to evaluate the prognostic value of different nutritional assessment scores in pancreatic surgery (NURIMAS Pancreas). Br J Surg 2017;104:1053–1062. 28369809 10.1002/bjs.10525

[5] Probst P , Haller S , Dörr‐Harim C , Bruckner T , Ulrich A , Hackert T , et al. Nutritional risk in major abdominal surgery: protocol of a prospective observational trial to evaluate the prognostic value of different nutritional scores in pancreatic surgery. JMIR Res Protoc 2015;4:e132. 26573991 10.2196/resprot.4567 PMC4704883

[6] Group* TVATPNCS . Perioperative total parenteral nutrition in surgical patients. N Engl J Med 1991;325:525–532. 1906987 10.1056/NEJM199108223250801

[7] Reilly HM , Martineau JK , Moran A , Kennedy H . Nutritional screening—evaluation and implementation of a simple Nutrition Risk Score. Clin Nutr 1995;14:269–273. 16843942 10.1016/s0261-5614(95)80063-8

[8] Kondrup J , Allison SP , Elia M , Vellas B , Plauth M . Educational and Clinical Practice Committee, European Society of Parenteral and Enteral Nutrition (ESPEN). ESPEN guidelines for nutrition screening 2002. Clin Nutr 2003;22:415–421. 12880610 10.1016/s0261-5614(03)00098-0

[9] Detsky A , McLaughlin JR , Baker J , Johnston N , Whittaker S , Mendelson R , et al. What is subjective global assessment of nutritional status? J Parenter Enteral Nutr 1987;11:8–13. 10.1177/014860718701100108 3820522

[10] British Association for Parenteral and Enteral Nutrition The ‘MUST’ Explanatory Booklet. A Guide to the ‘Malnutrition Universal Screening Tool’ (‘MUST’) for Adults. 2003. http://www.bapen.org.uk/pdfs/must/must_explan.pdf. Accessed 30 August 2021.

[11] Rubenstein LZ , Harker JO , Salvà A , Guigoz Y , Vellas B . Screening for undernutrition in geriatric practice: developing the short‐form mini‐nutritional assessment (MNA‐SF). J Gerontol A Biol Sci Med Sci 2001;56:M366–M372. 11382797 10.1093/gerona/56.6.m366

[12] Kruizenga HM , Seidell JC , de Vet HCW , Wierdsma NJ , van Bokhorst‐de van der Schueren MAE . Development and validation of a hospital screening tool for malnutrition: the short nutritional assessment questionnaire (SNAQ). Clin Nutr 2005;24:75–82. 15681104 10.1016/j.clnu.2004.07.015

[13] Tammam JD , Gardner L , Hickson M . Validity, reliability and acceptability of the Imperial Nutritional Screening System (INSYST): a tool that does not require the body mass index. J Hum Nutr Diet 2009;22:536–544. 20002950 10.1111/j.1365-277X.2009.01004.x

[14] Cederholm T , Bosaeus I , Barazzoni R , Bauer J , Van Gossum A , Klek S , et al. Diagnostic criteria for malnutrition—an ESPEN Consensus Statement. Clin Nutr 2015;34:335–340. 25799486 10.1016/j.clnu.2015.03.001

[15] Longford NT . A fast scoring algorithm for maximum likelihood estimation in unbalanced mixed models with nested random effects. Biometrika 1987;74:817–829.

[16] Kovacevich DS , Boney AR , Braunschweig CL , Perez A , Stevens M . Nutrition risk classification: a reproducible and valid tool for nurses. Nutr Clin Pract 1997;12:20–25. 9197791 10.1177/011542659701200120

[17] Neoptolemos JP , Stocken DD , Friess H , Bassi C , Dunn JA , Hickey H , et al. A randomized trial of chemoradiotherapy and chemotherapy after resection of pancreatic cancer. N Engl J Med 2004;350:1200–1210. 15028824 10.1056/NEJMoa032295

[18] Oettle H , Neuhaus P , Hochhaus A , Hartmann JT , Gellert K , Ridwelski K , et al. Adjuvant chemotherapy with gemcitabine and long‐term outcomes among patients with resected pancreatic cancer: the CONKO‐001 randomized trial. JAMA ‐ J Am Med Assoc 2013;310:1473–1481. 10.1001/jama.2013.279201 24104372

[19] Yamada D , Eguchi H , Asaoka T , Tomihara H , Noda T , Wada H , et al. The basal nutritional state of PDAC patients is the dominant factor for completing adjuvant chemotherapy. Surg Today 2017;47:1361–1371. 28421348 10.1007/s00595-017-1522-x

[20] Schnelldorfer T , Adams DB , Fink AS , Berhman SW . The effect of malnutrition on morbidity after surgery for chronic pancreatitis. Am Surg 2005;71:466–473. 16044924

[21] Sierzega M , Niekowal B , Kulig J , Popiela T . Nutritional status affects the rate of pancreatic fistula after distal pancreatectomy: a multivariate analysis of 132 patients. J Am Coll Surg 2007;205:52–59. 17617332 10.1016/j.jamcollsurg.2007.02.077

[22] La Torre M , Ziparo V , Nigri G , Cavallini M , Balducci G , Ramacciato G . Malnutrition and pancreatic surgery: prevalence and outcomes. J Surg Oncol 2013;107:702–708. 23280557 10.1002/jso.23304

[23] Kanda M , Fujii T , Kodera Y , Nagai S , Takeda S , Nakao A . Nutritional predictors of postoperative outcome in pancreatic cancer. Br J Surg 2011;98:268–274. 20960457 10.1002/bjs.7305

[24] von Haehling S , Morley JE , Coats AJS , Anker SD . Ethical guidelines for publishing in the journal of cachexia, sarcopenia and muscle: update 2019. J Cachexia Sarcopenia Muscle 2019;10:1143–1145. 31661195 10.1002/jcsm.12501 PMC6818444

