# Supplementary material for: Prospective trial to evaluate the prognostic value of different nutritional assessment scores for survival in pancreatic ductal adenocarcinoma (NURIMAS Pancreas SURVIVAL)
Source: J Cachexia Sarcopenia Muscle. 2021 Sep 20;12(6):1940–7. doi: 10.1002/jcsm.12796 (PMC8718045; doi:10.1002/jcsm.12796)
Supplement: Supplementary file 1 — Table S1. Items included in nutritional assessment scores. [file JCSM-12-1940-s001.docx]

| **Nutritional assessment score** | **Factors included** | **Stratification system*** |
| --- | --- | --- |
| ESPEN malnutrition criteria (ESPEN)^14^ | BMI, weight loss, fat free mass index | Normal/ Malnourished |
| Imperial Nutritional Screening System I (INSYST 1)^13^ | Weight loss, ability to eat | Not at risk/ At risk |
| Imperial Nutritional Screening System II (INSYST 2)^13^ | Diet, weight loss, other risk factors (e.g. cancer or infection) | Green/ Amber/ Red |
| Malnutrition Universal Screening Tool (MUST)^10^ | Weight loss, BMI, ability to eat | Low (0) / Medium (1) / High (≥2) |
| Mini Nutritional Assessment (MNA)^11^ | Ability to eat, weight loss, mobility, stress factors, neuropsychological problems, BMI, place patient lives, medication, pressure sores or skin ulcers, number of meals, diet, mode of feeding, self view of nutritional and health status, physical examination (mid-arm and calf circumference) | Normal (24-30) / At risk (17-23,5) / Malnourished (0-17) |
| Mini Nutritional Assessment SF (MNA-SF)^11^ | Ability to eat, weight loss, mobility, stress factors, neuropsychological problems, BMI, calf circumference | Normal (12-14) / At risk (8-11) / Malnourished (0-7) |
| Nutritional Risk Index (NRI)^6^ | Albumin, weight loss | Normal (>100) / Mild (97,5-100) / Moderate (83,5-97,5)/ Severe (< 87,5) |
| Nutritional Risk Screening Score (NRS)^7^ | Weight loss, BMI, appetite, ability to eat, stress factors (e.g. major surgery) | Low (0-3) / Moderate (4-6) / High (7-15) |
| Nutritional Risk Screening Score 2002 (NRS 2002)^8^ | Weight loss, BMI, ability to eat, stress factors (e.g. major surgery), age | Not at risk (0-2) / At risk (3-7) |
| Short Nutritional Assessment Questionnaire (SNAQ)^12^ | Weight loss, appetite, nutritional supplements | Low (<2) / Moderate (2) / Severe (≥3) |
| Subjective Global Assessment (SGA)^9^ | Weight loss, ability to eat, GI symptoms (e.g. nausea), physical ability, disease, physical examination (e.g. edema) | No/ Moderate/ Severe |

** For scores with more than 2 modes, only those assigned to the highest-risk class were defined as being “at risk for malnutrition” while all other categories were classified as “not at risk for malnutrition”*
